# Characteristics and Results of the Treatment of Multiple Myeloma in the Subject under the Age of 65 at the University Hospital of Yopougon in Abidjan, Côte d'Ivoire

**DOI:** 10.1155/2013/583051

**Published:** 2013-12-26

**Authors:** Diebkilé Aïssata Tolo, Duni Sawadogo, Danho Clotaire Nanho, Boidy Kouakou, N'Dogomo Méité, Roméo Ayémou, Paul Kouéhion, Mozart Konan, Yassongui Mamadou Sékongo, Emeraude N'Dhatz, Ismaël Kamara, Alexis Silué, Kouassi Gustave Koffi, Ibrahima Sanogo

**Affiliations:** Department of Clinical Hematology, Yopougon Teaching Hospital, P.O. Box 632, Abidjan 21, Cote d'Ivoire

## Abstract

We retrospectively studied 30 cases of multiple myeloma in patients under the age of 65, diagnosed from 1991 to 2005 in the clinical hematology department of the University Hospital of Yopougon that is a hospital incidence of 2.9 cases/year. The age of patients ranged from 34 to 64 years, with a mean age of 49 years and a sex ratio of 1.73. The professional activity was variable with 3% of radiographers and 10% of farmers. Clinically, the dominant sign was bone pain in 83% of cases. Myeloma was secretory in 93% of cases. It was Ig G-type in 86%, kappa-type in 66% of cases. 86% of patients were anemic, 20% had creatinine >20 mg/L, and 10% had serum calcium >120 mg/L. Geodes were found in 80% of cases. 53% were at stage III of DURIE and SALMON. Complications were infectious (33%), renal (20%), and hemorrhagic (7%). Chemotherapy regimens were VAD (10%), VMCP (30%), and VMCP/VBAP (60%) with 47% of partial responses, 33% of stable disease, and 7% of very good quality partial responses. The outcome developed towards death in 37% and causes of death were renal in 46% of cases. The median survival was only 5.1 months.

## 1. Introduction

Multiple myeloma or Kahler disease is a malignant proliferation of an abnormal plasma cell clone secreting a complete or incomplete immunoglobulin. It, respectively, accounts for 10% and 20% of malignant hemopathies in the Caucasian and Black American. It is a condition of the subject of more than 50 years of age. Its incidence increases with age: 5 per 100,000 individuals at the age of 60 and 20 per 100,000 individuals at the age of 80. The average age at diagnosis is 64 years. It is slightly more common in men than in women [1].

Positive diagnosis of multiple myeloma is not always easy. It requires a combination of clinical arguments (general condition, bone syndrome), biological arguments (study of marrow, study of protein in the blood and urine, hemoglobin level, serum calcium, and creatinine), and radiological arguments (X-ray of the skeleton) [2].

The outcome is punctuated by multiple complications such as bone, renal, infectious, metabolic, neurological, hemorrhagic complications, and cachexia.

Initially the prognostic classification was based on that of DURIE and SALMON but currently it is the ISS (International Staging System) with two parameters albumin and *β*2 microglobulin, which is used. In addition, some cytogenetic abnormalities such as del (13q), t(11; 14), t(4; 14), t(14; 16), and del (17p) have a major prognostic value. They have poor prognosis [3–6]. Mortality is 4.1/100,000 inhabitants/year in Europe [3].

Therapeutically, the treatment is not recommended for patients at stage I of DURIE and SALMON; on the other hand for stages II and III, treatment will be established taking into account the patient's age [3]. If, in developed countries, patients under the age of 65 receive intensive treatment with bone marrow graft particularly, this is not the case in Côte d'Ivoire. What were the results of treatment in these patients in our context of exercise and what were the features of their myeloma?

## 2. Patients and Methods

Our study was carried out in the Clinical Hematology Department of the University Hospital of Yopougon in Côte d'Ivoire. It was retrospective and descriptive and focused on the records of patients hospitalized for symptomatic multiple myeloma, diagnosed in the period from January 1991 to August 2005 by myelogram, the study of protein in the blood and urine, hemoglobin level, serum calcium, creatinine, skeletal X-ray according to SWOG criteria (Southern Western Oncology Group) and ROTI (multiple myeloma-related organ or tissue impairment: elevation of serum calcium, anemia, kidney failure, and bone lesions) [6, 7], and who had received chemotherapy. Thirty patients were included in the study. The patients with asymptomatic multiple myeloma and monoclonal gammopathies of undetermined significance were not selected. Each medical record was operated using an individual survey form with collection of epidemiological parameters (age, sex, occupation, and socioeconomic status), clinical parameters (performance status, bone pain), biological parameters (bone marrow plasma cells, type of monoclonal immunoglobulin, type of light chain, Bence Jones proteinuria, hemoglobin level, serum calcium, and creatinine), radiological parameters (geodes, demineralization, bone tumors, and bone fractures), evolutionary parameters (classification of DURIE and SALMON, progressive complications, and death), and therapeutic parameters according to the index of standardized generalized response (complete remission or CR, very good partial response or VGPR, partial response or PR, stable disease, progression, and relapse) (Table 2).

The socioeconomic status was assessed using indirect criteria: habitat type, occupation, ability to meet the cost of prescriptions, and number of dependent children.

CR corresponds to the negativity of monoclonal immunoglobulin in the blood and urine and to bone marrow plasma cells <5%.

VGPR corresponds to serum monoclonal immunoglobulin decreased by 90% and to urinary immunoglobulin <100 mg/24 H.

PR corresponds to serum monoclonal immunoglobulin decreased by 50%, urinary monoclonal immunoglobulin decreased by 90%, and free light chains decreased by 50%.

Stable disease corresponds to the lack of criteria for CR, VGPR, and PR.

Progression criteria are 25% increase of serum or urine monoclonal immunoglobulin or free light chains assay, an increase of 10% of the bone marrow plasma cells, appearance of new bone or extra bone lesions, and a level of calcium greater than 2.65 mmol/L.

The criteria for relapse are appearance of new bone or extra bone lesions, a level of serum calcium greater than 2.65 mmol/L, hemoglobin level lowering to 2 g/dL, and a level of creatinine greater than 20 mg/L (177 *μ*mol/L); the monoclonal component alone is not taken into account for relapse [7].

Therapeutically, three chemotherapy regimens were used in our patients (VAD: vincristine, adriamycin, dexamethasone protocol, VMCP: vincristine, melphalan, cyclophosphamide, prednisone protocol, VMCP/VBAP: VMCP/vincristine, carmustine, adriamycin, prednisone alternate protocol) in spaced treatments of 4 to 6 weeks associated with adjuvant therapies (bisphosphonates, antiemetics, and potassium).

**Figure 1:**
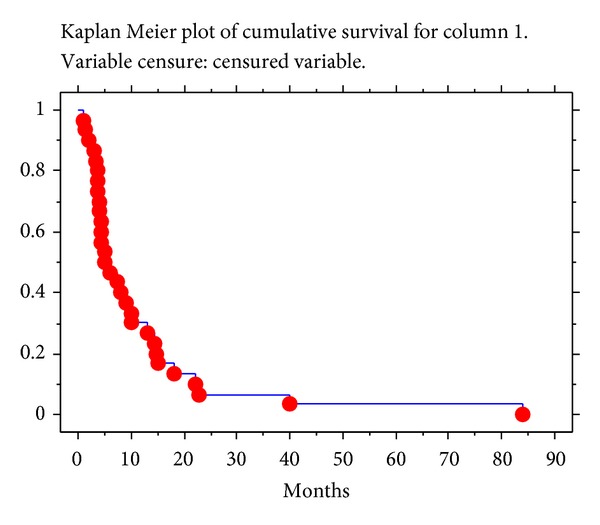
Overall survival curve.

Data were analyzed using Epi-Info software version 6.04b, Statview. The calculation of overall survival was performed according to the Kaplan Meier method with the existence in the record of an inclusion date (admission date) and a date of final assessment (date of death or the latest information mentioned in day, month, and year (dd-mm-yyyy)) (Figure 1).

## 3. Results

From January 1991 to August 2005, the diagnosis of symptomatic multiple myeloma was made in 30 patients under the age of 65, out of a total number of 44 cases of myeloma. The epidemiological, clinical, and biological features of these patients are summarized in Table 1.

## 4. Discussion

This study of multiple myeloma was carried out because very few data exist on the international level concerning the myeloma of the Ivorian.

The Clinical Hematology Department of the University Hospital of Yopougon is the only center of therapeutic management of hematological malignancies.

Out of a total of 44 cases of symptomatic multiple myeloma diagnosed from 1991 to 2005, in 15 years, we had a hospital incidence of 2.9 cases/year. The prevalence increased steadily with age: 10% among patients under the age of 40, 33% among 40–51-year-old patients, and 57% among 52–64-year-old patients. In addition, patients under the age of 65 accounted for 68.2% of all of our myelomas. Thus, the predominance of young patients under the age of 65 is one of the epidemiological features of our myelomas, whereas, in Europe, 40% of patients are under the age of 65 and less than 2% are under the age of 40 [8]. The average age of onset in Europe is typically around 65 to 70 years [6, 8]. The average age of our study population was 49 years, with extremes of 34 and 64. Our low average age is due to our type of sampling (study population aged under 65), and also due to the age pyramid of the African populations. However, all authors agree that myeloma is rare before the age of 40 and that it is predominant among the male subject, as shown in our study [4, 6, 9].

In our study population, the occupation was very variable, ranging from executives to the informal sector and housewives. Exposure to ionizing radiation is the only established risk factor. This risk was found in one patient who was a radiographer. No case of family myeloma was noted. Some authors mentioned environmental factors (pesticides, herbicides, and fertilizers), especially among farmers who accounted for 10% of our study population [1].

Clinically, a bone syndrome was found in 83% of cases. It was characterized by bone pains associated or not with a bone tumor (3%) or a fracture (33%). Thus, bone pain was the dominant clinical sign in our patients, not relieved by conventional analgesics. The performance status was 2 or 3 in 80% of cases, most often associated with asthenia due to anemia or other evolutionary complications particularly recurrent infections (33%), renal failure (20%), and bleeding (7%). Infections were urogenital in 5 cases, pulmonary in 2 cases, ENT in 1 case, mucocutaneous in 1 case, and musculoskeletal in 1 case [1, 6].

Paraclinically, anemia was found in 86% of cases. This anemia was severe <8 g/dL in 43% of cases in our study population. In the literature we have, anemia is observed in 40–73% of cases [6, 9]. Bone marrow plasma cells were between 10 and 30% in 60%, the monoclonal immunoglobulin of type G in 86%, the kappa light chains in 66%, and secretory myeloma in 93%. Creatinine was <20 mg/L in 80% and serum calcium <120 mg/L in 90%. Radiological signs were dominated by geodes (80%) [10, 11]. No patient received magnetic resonance imaging (MRI) or serum determination of free light chains or cytogenetic examination for the detection of chromosomal abnormalities.

In terms of outcome, myeloma was at stage II of DURIE and SALMON in 47% of cases and at stage III in 53% of cases. The ISS (International Staging System) could not be assessed because the determination of *β*2 microglobulin could not be performed in the majority of patients.

Therapeutically, we only resorted to chemotherapy associated with bisphosphonates in our patients because we did not have bone marrow graft. Three chemotherapy regimens were used: VAD protocol (10%), VMCP protocol (30%), and VMCP/VBAP alternate protocol (60%). These treatments resulted in 47% of partial responses, 7% of very good partial responses, 33% of stable disease, and 13% of progression. No case of complete remission or relapse was recorded [7]. The absence of complete remission is due to the lack of intensification and the poor compliance by the fact that most patients do not complete their treatment plan, mostly for financial problems, given that 84% of our patients were of low socioeconomic conditions.

Four parameters have influenced our responses to treatment:

- the absence of bone marrow transplant and new drugs for multiple myeloma (Bortezomib, IMiDs),
- the large number of patients lost to followup (43%),
- the fact that the Black has a mortality rate higher than the Caucasian [12],
- the problems of access to antimitotics: patients who are mostly of low socioeconomic status are obliged to buy medicines at exorbitant prices for their treatment.

Despite this, our results can be considered acceptable since our complete remission rate was 47%. This rate is close to that of Alexanian et al. in the US and Wan in China who have obtained, respectively, 55% and 59.09% with a protocol including vincristine, VAD [13, 14].

Our challenge that is to improve our response rate will probably be taken up because the service has new treatments for multiple myeloma.

Concerning the outcome of patients, 13 were lost to followup, that is, 43%, 11 had died, that is, 37%, and 6 were still alive and on treatment, that is, 20%. We have no data concerning the monitoring of the cohort of 6 patients alive from 2005 to 2013. This is the reason why the maximum survival time was 84 months in our Kaplan Meier curve. So there is no median followup of this cohort. The causes of death were renal in 47% and infectious in 27%, related to the progression of the disease in 27%. The median overall survival was 5.1 months with extremes ranging from 0 to 84 months. The probability of survival at 6 months was 51% and at 3 years 6.7%. But it should be noted that currently the gold standard in subjects under the age of 65 is the VTD (velcade, thalidomide, dexamethasone) protocol, and we have this protocol at our disposal since October 2010. But only well-off patients have access to it, given its relatively high cost. But in our regions, there is no international clinical trial for multiple myeloma at the moment.

## Figures and Tables

**Table 1:** Epidemiological, clinical, and biological features of patients.

Variables	Numbers (%)
Age (years): average and extremes: 49 (34–64)	
<40	3 (10)
40–51	10 (33)
52–64	17 (57)
Sex: sex ratio: 1.73	
Male	19 (63)
Female	11 (37)
Professional occupations	
Executives	8 (27)
Housewives	4 (13)
Informal sector	4 (13)
Others	14 (47)
Socioeconomic status	
Low	8 (27)
Average	17 (57)
High	5 (16)
Performance status	
0 and 1	3 (10)
2 and 3	24 (80)
4	3 (10)
Bone syndrome	
Bone pains	25 (83)
Bone tumors	1 (3)
Fractures	10 (33)
Bone marrow plasma cells	
10 to 30%	18 (60)
>30	12 (40)
Whether secretory or not	
Secretory	28 (93)
Nonsecretory	2 (7)
Type of monoclonal immunoglobulin	
Ig G	26 (86)
Ig A	2 (7)
Light chains myeloma	2 (7)
Type of light chain	
Kappa	20 (86)
Lambda	2 (7)
Undetermined	2 (7)
Hemoglobin level (g/dL)	
<8	13 (43)
8–12	13 (43)
>12	4 (14)
Serum creatinine (mg/L)	
<20	24 (80)
>20	6 (20)
Calcium (mg/L)	
<120	27 (90)
>120	3 (10)
Bence Jones proteinuria (mg/24 H)	
<12	24 (80)
>12	6 (20)

Other professional activities: radiographer, farmers, policeman, traders, and teachers.

**Table 2:** Radiological, evolutional, and therapeutic features.

Variables	Numbers (%)
Radiological signs	
Geodes	24 (80)
Demineralization	12 (40)
Bone tumors	1 (3)
Fractures	10 (33)
Seats of fractures	
Femur	6 (20)
Humerus	2 (7)
Rib	1 (3)
Tibia	1 (3)
Stage of DURIE and SALMON	
Stage I	0 (0)
Stage II	14 (47)
Stage III	16 (53)
Complications	
Acute renal failure	2 (7)
Chronic renal failure	2 (7)
Infectious	10 (33)
Hemorrhagic	2 (7)
Therapeutic protocols	
VAD	3 (10)
VMCP	9 (30)
VMCP/VBAP	18 (60)
Therapeutic responses	
CR	0 (0)
VGPR	2 (7)
PR	14 (47)
Stable disease	10 (33)
Progression	4 (13)
Relapse	0 (0)
Outcome	
Living and on treatment	6 (20)
Lost to followup	13 (43)
Dead	11 (37)
Causes of death	
Progression	3 (27)
Renal complication	5 (46)
Infectious complication	3 (27)
Survival (median and extreme values in months)	5.1 (0–84)

## References

[1] Chaubert AB, Delacretaz F, Schmidt PM (2005). Myélome multiple. *Schweizerische Medical Forum*.

[2] Bataille R (1996). Myélome multiple: traitements symptomatiques et antitumoraux. *Encycl Méd Chir*.

[3] Harousseau J-L, Dreyling M (2010). Multiple myeloma: ESMO clinical practice guidelines for diagnosis, treatment and follow-up. *Annals of Oncology*.

[4] Dong WJ-, Chang-Ki M, Kyungja H (2013). Impact of genetic abnormalities on the prognoses and clinical parameters of patients with multiple myeloma. *Annals of Laboratory Medicine*.

[5] Reece DE (2009). Recent trends in the management of newly diagnosed multiple myeloma. *Current Opinion in Hematology*.

[6] Palumbo A, Cerrato C (2013). Diagnosis and therapy of multiple myeloma. *The Korean Journal of Internal Medicine*.

[7] Bird JM, Owen RG, D’Sa S (2011). Guidelines for the diagnosis and management of multiple myeloma 2011. *British Journal of Haematology*.

[8] Oranger A, Carbone C, Grano M (2013). Cellular mechanisms of multiple myeloma bone disease. *Clinical and Developmental Immunology*.

[9] Dispenzieri A, Kyle RA (2005). Multiple myeloma: clinical features and indications for therapy. *Best Practice and Research*.

[10] Kyle RA, Gertz MA, Witzig TE (2003). Review of 1027 patients with newly diagnosed multiple myeloma. *Mayo Clinic Proceedings*.

[11] Shaw GR (2006). Nonsecretory plasma cell myeloma—becoming even more rare with serum free light-chain assay: a brief review. *Archives of Pathology and Laboratory Medicine*.

[12] Pulte D, Redaniel MT, Brenner H, Jansen L, Jeffreys M Recent improvement in survival of patients with multiple myeloma: variation by ethnicity.

[13] Alexanian R, Barlogie B, Tucker S (1990). VAD-based regimens as primary treatment for multiple myeloma. *American Journal of Hematology*.

[14] Wan J (2013). Therapeutic efficacy analysis of VD regimen and VAD regimen for multiple myeloma. *Zhongguo Shi Yan Xue Ye Xue Za Zhi*.

